# Trypan Blue and Endoillumination-Assisted Phacoemulsification in a Patient With Advanced Keratoglobus

**DOI:** 10.7759/cureus.56265

**Published:** 2024-03-16

**Authors:** Yumna F Kamal, Mazen Alzahrani, Halah Bin Helayel, Sami T Hameed

**Affiliations:** 1 Ophthalmology, Ministry of National Guard Hospital, Jeddah, SAU; 2 Ophthalmology, East Jeddah Hospital, Jeddah, SAU; 3 Ophthalmology, King Khaled Eye Specialist Hospital, Riyadh, SAU

**Keywords:** cataract, keratoglobus, surgical techniques, trypan blue, endoillumination-assisted phacoemulsification

## Abstract

Keratoglobus is a rare subset of noninflammatory corneal ectasia, which is a group of disorders characterized by corneal thinning, projection, and scarring. Patients with keratoglobus commonly present with poor vision. A case of advanced keratoglobus was managed by a modified phacoemulsification surgical technique using endoillumination and capsular staining with trypan blue. In this case, we present a 54-year-old man with keratoglobus. In January 2023, a modified phacoemulsification surgical technique using endoillumination was described with a video in a patient with bilateral corneal opacification, neovascularization, significant peripheral thinning, and moderate to severe corneal opacity in which cataract surgery had to be performed alone without considering penetrating keratoplasty. Postoperatively, the patient was doing well with no leaks. We may conclude that this method allows for better visualization during surgery and decreases the risk of intraoperative complications due to poor visualization in patients with severe corneal opacity.

## Introduction

The most commonly preferred technique for cataract extraction nowadays in developed countries is phacoemulsification. Other techniques, such as extracapsular cataract extraction (ECCE) and small incision cataract surgery (SICS), are also employed in certain cases, especially where phacoemulsification is deemed challenging such as cases with significant corneal opacity, hard cataract, and endothelial cell dysfunction [1]. In cases with absent red reflex due to mature cataract, for example, successful completion of continuous curvilinear capsulorhexis (CCC) can be achieved by using trypan blue (VisionBlue^®^), as it helps in improving visualization of the anterior capsule, thereby reducing the risk of complications. Furthermore, in response to the possible concerning side effects of its use, a study showed that trypan blue was not harmful to the corneal endothelium when applied during cataract surgery, especially if washed out quickly [2]. Also, endoillumination is another tool that can be utilized when visualizing anterior chamber structures, and the lens is not optimal intraoperatively. The use of endoillumination and capsular staining for cases with impaired visualization due to keratoglobus is rarely described in the literature. In this report, we present a patient with advanced keratoglobus, cataract, and moderate to severe corneal opacity in which cataract surgery had to be performed alone without considering penetrating keratoplasty (PKP).

## Case presentation

**Figure 1:**
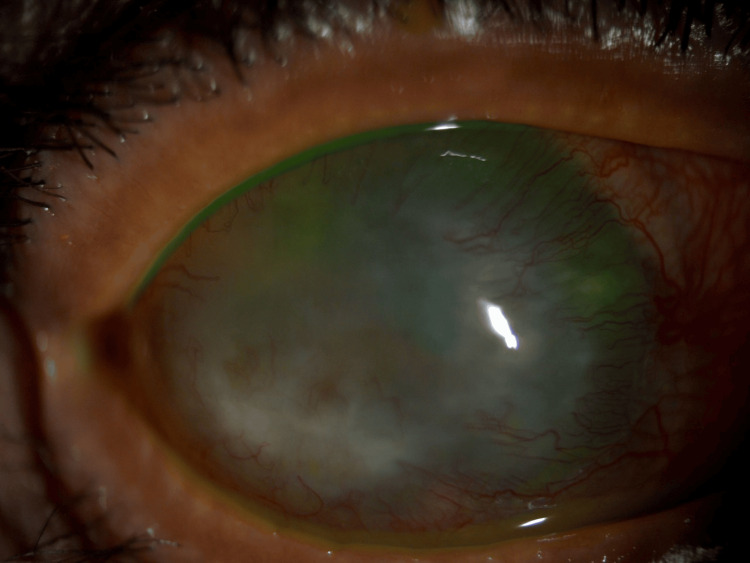
External photo of the eye showing keratoglobus with moderate to severe corneal opacity

A 54-year-old male patient who is a known case of diabetes mellites, hypertension, and advanced keratoglobus presented to the Ophthalmology clinic complaining of poor vision in both eyes. Upon examination of the eye, the visual acuity was hand motion on the right eye and 1/200 on the left eye. With scleral contact lens correction, visual acuity reached 20/125. His intraocular pressure (IOP) was 11/13. His conjunctiva and sclera were quiet bilaterally. He had bilateral corneal opacification with neovascularization and significant peripheral thinning. There was no perforation or leak. The anterior chamber (AC) was deep and quiet, and the lens was cataractous. The initial impression was advanced keratoglobus with severe corneal thinning bilaterally (Figure 1). A B-scan was requested to check for posterior pole pathology due to poor visualization of the fundus, and it showed no pathology. After obtaining informed consent and explaining the possible visual prognosis of his condition, the patient underwent phacoemulsification in the right eye, as per his preference.

Topography and pachymetry

**Figure 2:**
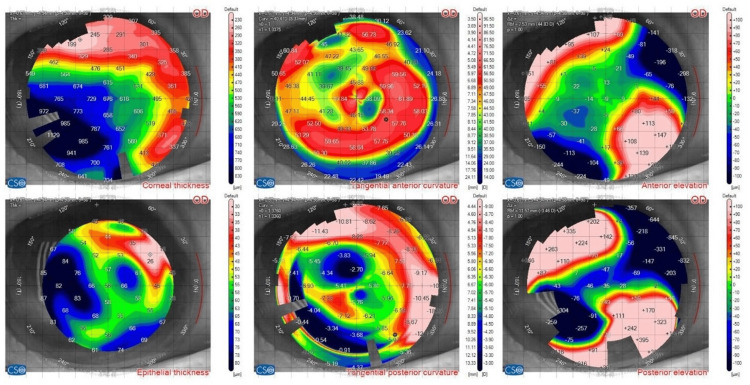
Pachymetry

**Figure 3:**
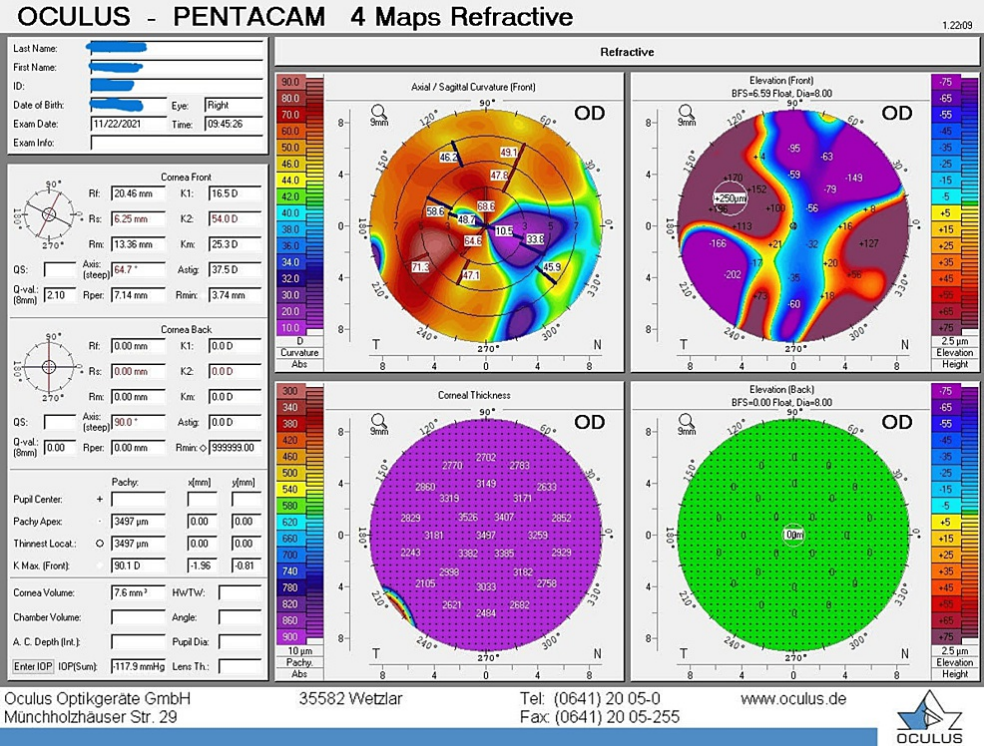
Topography

Pachymetry and topography values are shown in Figures 2, 3, respectively.

Surgical technique

The eye was prepared in a standard sterile manner. First, paracenteses were performed using a super sharp blade. Viscoelastic material (OVDs) was injected into the anterior chamber. Endoillumination light was used to assist in AC visualization and then iris hooks were placed. After that, the OVDs were aspirated. VisionBlue was used to stain the anterior capsule and then was washed out with a balanced salt solution (BSS). OVDs were injected into the AC. Anterior capsulotomy was performed in a CCC manner. A conjunctival peritomy was then performed, followed by gentle wet field cautery to provide hemostasis. Later, a 3 mm scleral flap was created, and then the sclerocorneal wound was entered. Hydrodissection was carried out, followed by phacoemulsification using the divide and conquer technique. The entire surgical procedure was uneventful except for a posterior capsule rupture, which was noticed after the complete removal of the nucleus. Triamcinolone was used to check for the vitreous presence near the wound and in the AC. Adequate anterior vitrectomy was performed, and the remaining cortical material was aspirated. We opted not to implant an intraocular lens (IOL) as the power of the IOL calculated in this case was +2 using the IOL Master 500 and Holladay 1 formula. Finally, all wounds were closed using 10-0 nylon, checked with fluorescein, and showed no leak. The conjunctiva was closed using 8-0 vicryl; Video 1 demonstrates the surgical steps. Postoperatively, moxifloxacin eye drops were prescribed four times per day for two weeks and prednisolone for 1 month in a tapering schedule starting with four times daily in the first week. On the first postoperative day, the cornea was clear with uncorrected visual acuity of 20/400. Upon slit-lamp examination, no leak was noticed. The AC was deep with a good red reflex. A follow-up appointment was given in two weeks to remove sutures. As no intraocular lens was inserted, the vision remained 20/400. The patient did not improve with glasses and refused contact lenses.

**Video 1:** Trypan blue and endoillumination-assisted phacoemulsification in a patient with advanced keratoglobus

## Discussion

Keratoglobus compromises a rarer subset of noninflammatory corneal ectasia, which is a group of disorders characterized by corneal thinning, projection, and scarring [3]. Patients with keratoglobus commonly present with poor vision. A study by Rathi et al. showed that patients with keratoglobus had visual acuity of counting fingers close to the face to no light perception in 50% of pediatric and 37.74% of adult patients [4]. Most cases of keratoglobus can be managed conservatively with glasses or contact lenses. In addition to improving vision, spectacles also provide a protective role, as these patients are more susceptible to corneal rupture following corneal trauma. Management may resort to the need for a corneal graft. Lamellar keratoplasty and partial thickness corneoscleroplasty followed by PK are surgeries that may be done to better graft survival and reduce rejection as well as maintain globe integrity [5,6]. When there is both a cataract and significant corneal opacity, combined PKP and lens extraction can be performed. However, in this case, where there are several risk factors, such as advanced keratoglobus, severe thinning, significant scarring, and vascularization, there is a particularly increased risk of corneal leak and rejection [7]. Also, a PKP in this patient would likely have required long-term immunosuppressant medications to reduce the risk of rejection, which would be undesirable for older individuals with comorbidities. Therefore, we opted to perform phacoemulsification surgery in this case; however, in this scenario, performing phacoemulsification is not without challenges. Therefore, modifications of the routine technique are crucial to reduce the risk of intraoperative complications and ensure smooth recovery postoperatively [8]. We utilized trypan blue staining and endoillumination to improve intraoperative visualization in this case. This technique is similar to the one employed by Yamamoto et al. in which a red reflex can still be obtained and lens materials remain visible [9]. In addition, our technique included the use of a scleral flap for the main wound to reduce the risk of corneal leak. A similar approach has been reported by Terubayashi et al. for severe scleral thinning [10].

## Conclusions

As a result, we suggest that trypan blue and endoillumination-assisted phacoemulsification is a useful method to obtain better visibility of anterior chamber structures, especially in cases of keratoglobus and cataract where PKP or ECCE are associated with an increased risk of a postoperative leak.
